# Computational Analysis of Phosphopeptide Binding to the Polo-Box Domain of the Mitotic Kinase PLK1 Using Molecular Dynamics Simulation

**DOI:** 10.1371/journal.pcbi.1000880

**Published:** 2010-08-12

**Authors:** David J. Huggins, Grahame J. McKenzie, Daniel D. Robinson, Ana J. Narváez, Bryn Hardwick, Meredith Roberts-Thomson, Ashok R. Venkitaraman, Guy H. Grant, Mike C. Payne

**Affiliations:** 1 TCM Group, Cavendish Laboratory, University of Cambridge, Cambridge, United Kingdom; 2 Cambridge Molecular Therapeutics Programme, Hutchison/MRC Research Centre, University of Cambridge, Cambridge, United Kingdom; 3 Schrödinger, Camberley, United Kingdom; 4 Unilever Centre for Molecular Informatics, The University Chemical Laboratory, University of Cambridge, Cambridge, United Kingdom; National Cancer Institute, United States of America

## Abstract

The Polo-Like Kinase 1 (PLK1) acts as a central regulator of mitosis and is over-expressed in a wide range of human tumours where high levels of expression correlate with a poor prognosis. PLK1 comprises two structural elements, a kinase domain and a polo-box domain (PBD). The PBD binds phosphorylated substrates to control substrate phosphorylation by the kinase domain. Although the PBD preferentially binds to phosphopeptides, it has a relatively broad sequence specificity in comparison with other phosphopeptide binding domains. We analysed the molecular determinants of recognition by performing molecular dynamics simulations of the PBD with one of its natural substrates, CDC25c. Predicted binding free energies were calculated using a molecular mechanics, Poisson-Boltzmann surface area approach. We calculated the per-residue contributions to the binding free energy change, showing that the phosphothreonine residue and the mainchain account for the vast majority of the interaction energy. This explains the very broad sequence specificity with respect to other sidechain residues. Finally, we considered the key role of bridging water molecules at the binding interface. We employed inhomogeneous fluid solvation theory to consider the free energy of water molecules on the protein surface with respect to bulk water molecules. Such an analysis highlights binding hotspots created by elimination of water molecules from hydrophobic surfaces. It also predicts that a number of water molecules are stabilized by the presence of the charged phosphate group, and that this will have a significant effect on the binding affinity. Our findings suggest a molecular rationale for the promiscuous binding of the PBD and highlight a role for bridging water molecules at the interface. We expect that this method of analysis will be very useful for probing other protein surfaces to identify binding hotspots for natural binding partners and small molecule inhibitors.

## Author Summary

Cell division is a key biological process and imperfections in the process can lead to diseases like cancer. Polo-Like Kinase 1 (PLK1) is a protein kinase enzyme that controls cell division by interacting with many other proteins. Malfunction of PLK1 has been implicated in cancer. To understand how PLK1 interacts with so many other proteins, we created a three-dimensional model of PLK1 and simulated its dynamic nature. Analysis of the components of the binding affinity provided insight into how the binding specificity is achieved. We also employed a method of analysis that locates regions of the protein surface that are particularly important in controlling binding affinity. Our results not only provide a valuable tool that can be generally applied to analyzing the binding between protein surfaces, but also provide insights into how PLK1 works to control cell division by binding to specific partners. In the future, these analyses could help to design drugs that block the interaction between PLK1 and its partners to block cell division for the treatment of diseases like cancer.

## Introduction

Mitotic cell division involves a tightly orchestrated series of events that precisely segregate an equal complement of chromosomes to two daughter cells. Abnormalities in mitosis generate aneuploid cells containing an unequal distribution of chromosomes, which may represent a starting point for the genesis of cancer. The polo-like kinase 1 (PLK1) is an important of mitosis, working at different steps to facilitate mitotic entry, progression through the stages of chromosome segregation, and finally, mitotic exit [1]–[3]. To do so, PLK1 must phosphorylate a wide range of protein substrates, yet operate in a manner that is tightly controlled in space and time [4]. How these conflicting requirements for PLK1 activity are fulfilled during mitosis remains unclear. However, recent findings suggest that PLK1 activity is frequently mis-regulated in human cancers. Thus, PLK1 is overexpressed in a wide range of human tumours, with high expression levels often correlating with poor prognosis [5].

**Figure 1:**
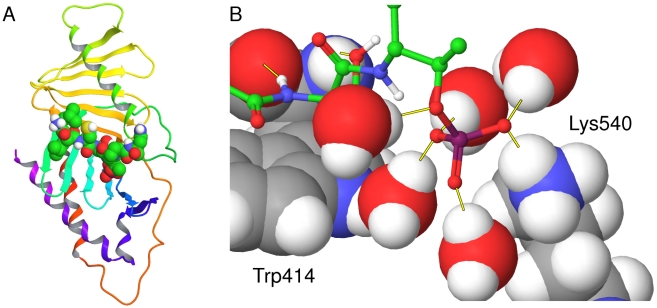
The PBD of PLK1 crystallised with the CDC25c phosphopeptide. The crystal structure of the PBD of PLK1 in complex with the CDC25c phosphopeptide LLCSpTPN from PDB ID 3BZI. (a) The protein represented as a ribbon diagram with the phosphopeptide displayed by atom-colored space filling (b) The binding site shown in more detail. The phosphopeptide is displayed as atom colored ball and sticks, interfacial water molecules are displayed in atom-colored CPK and residues Trp414 and Lys540 are named in black and displayed in atom-colored CPK. Key hydrogen bonds are displayed as yellow lines.

PLK1 consists of two distinct functional domains: an N-terminal kinase domain responsible for catalytic activity, and a C-terminal polo-box domain (PBD), which binds PLK1 target proteins. A flexible linker of approximately 50 amino acids joins these two domains together. The kinase activity leads to the phosphorylation and activation of a number of key mitotic proteins, notably Wee1, CDC25c, BubR1 and CyclinB1 [6]–[8]. Studies have established that the PBD is a phosphopeptide binding domain which binds to the consensus phosphopeptide sequence [Pro/Phe]-[Φ/Pro]-[Φ]-[Thr/Gln/His/Met]-Ser-[pThr/pSer]-[Pro/Φ], where Φ represents a hydrophobic residue [9]–[10]. Elia *et al* also identified a high-affinity synthetic phosphopeptide for PLK1, which has the sequence PMQSpTPL. However, at the majority of positions in this sequence, there is no particular preference for specific residues. This relatively broad specificity with respect to phosphopeptide binding allows PLK1 to bind a large set of phosphorylation-primed target proteins. A comprehensive proteomic analysis identified 622 potential binding partners of PLK1 [11] and at least 17 of these have been confirmed as binding partners [1]. Structural elucidation of the PBD has shown that it adopts a unique fold which forms a narrow groove into which phosphopeptides bind [9], [12]–[14]. The structure of the PBD of PLK1 from the Protein Data Bank (PDB) with PDBID 3BZI [10] can be seen in Figure 1. The exact mechanism for the function of the PBD has not been definitively determined, but the evidence suggests that it provides a scaffold where proteins can bind after they have been phosphorylated at other sites by priming enzymes like cyclin-dependent kinases [10]. It has been suggested that these bound proteins may then act as substrates for the kinase activity of PLK1 or may cause a conformational change allowing other substrates to bind to the kinase domain. Such regulatory mechanisms are found in other kinases, where interaction domains such as FHA, SH2, WW and 14-3-3 act as molecular switchboards, allowing interactions to occur with specific partners [15]. The available crystal structures identify Trp414, His538 and Lys540 as the key residues for phosphopeptide binding. The importance of these residues has been confirmed by cell-based experiments where their mutation abolishes PBD binding capacity [10], [16].

Considering the phosphopeptide sequence, initial work highlighted a striking selectivity for serine at the −1 position and slight selectivity for proline at the +1 position, but very little selectivity at any other position [9]. This lack of selectivity suggests that the phosphopeptide recognition site is highly promiscuous. Whilst this is unusual, it is consistent with PLK1's multiple functions throughout mitosis. However, the existence of such a large number of PBD-interacting phosphopeptides demands a molecular explanation. One striking feature of the PBD crystal structures generated to date is the nature of the interfacial contacts made between the PBD and bound phosphopeptide. Yun *et al* have recently crystallised a variety of short phosphopeptides complexes with the PBD [14]. Peptides as small as HSpTP and LHSpT were shown to bind to the PBD. This suggests that a large proportion of the binding energy is contributed by the core SpT motif. This suggestion is supported by analysis of the two complexes of the PBD with PMQSpTPL from PDBID 1UMW and LLCSpTPN from PDBID 3BZI. Both Gln and Cys residues form intermolecular interactions with the PBD at the −2 position and both Leu and Gln residues form intermolecular interactions at the +2 position [14]. The ability of very different residues to make contacts further supports the idea that the core SpT motif is the main determinant of binding. A second striking feature of the crystal structures of the PBD-phosphopeptide complexes is the large fraction of interactions between the phosphopeptide and the protein that are bridged by water molecules. For example, in PDBID 3BZI, there are 18 hydrogen bonding interactions between the PBD and the CDC25c phosphopeptide, 9 of which are bridged through water molecules. The number of interactions bridged by water molecules is significantly greater than in other phosphopeptide-binding proteins. For example, the phosphopeptide bound with the 14-3-3 protein from PDBID 1YWT [17] makes 13 hydrogen bonding interactions at the interface but only 1 through a water molecule. In other examples, the phosphopeptide bound with the SRC-SH2 domain from PDBID 2PIE [18] makes 16 hydrogen bonding interactions at the interface with only 4 through water molecules, and the phosphopeptide bound with the Rad53p-FHA1 domain from PDBID 1G6G makes 15 hydrogen bonding interactions, none of which are mediated by water molecules [19]. Therefore, bridging water molecules appear to play a specific and important role in PBD-phosphopeptide interactions.

The overall aim of the study was to explore the energetics and dynamics of PLK1 PBD interactions and probe the nature of the water molecules at the interface and how they affect binding. To assess the determinants of binding and gauge the role of water molecules in phosphopeptides binding to the PBD of PLK1, we performed molecular dynamics (MD) simulations to study the motion of atoms within the complexes. Crucially, this approach captures the dynamic aspects of interactions that are ignored by calculations performed on static systems [20]. It has also proved to be more accurate than other methods for estimating binding free energies [21]. The strengths of the interactions were estimated using a molecular mechanics, Poisson-Boltzmann surface area (MM-PBSA) approach [22]–[23]. This approach has been used in the past for analysing the role of water molecules at protein-protein interfaces [24]. The determinants of affinity and the importance of water molecules at peptide binding interfaces have also been explored using MD simulations, for the SRC SH2 domain [25] and at a small molecule binding interface for the GRB2 SH2 domain [26]. In addition, we assessed binding energy contributions from discrete residues within the phosphopeptide chain in an attempt to provide an energetic framework which explains the ability of PBD to accommodate such a wide range of phosphopeptide ligands and, therefore, to function with such a diverse range of targets throughout mitosis. Lastly, we employed inhomogeneous fluid solvation theory to predict the enthalpy and entropy of hydration sites on the surface of the apo protein and the phosphopeptide complex in order to understand the importance of water molecules in mediating the intermolecular interactions. This understanding allows us to elucidate the mechanisms controlling affinity and specificity in this system, providing biologically relevant information and facilitating drug development efforts.

## Materials and Methods

We performed MD simulations of the PBD in the phosphopeptide-bound complex, the peptide-bound complex and the apo state. The phosphopeptide-bound structure in complex with the CDC25c sequence LLCSpTPN was taken from PDB ID 3BZI at 2.10 Å resolution [10]. The crystal structure is taken from a protein construct containing PLK1 residues 367–603, of which residues 371–593 have assigned density. This version of the PBD is more amenable for crystallisation and still retains the crucial features which are responsible for substrate binding and specificity [10]. We did not use the peptide-bound structure in complex with the CDC25c sequence LLCSTPN from PDB ID 2OJX as it has a resolution of 2.85 Å and residues 489–506 could not be assigned from the density. Instead, we generated the peptide-bound complex by deletion of the phosphate group from the phosphopeptide-bound complex from PDBID 3BZI. The apo state was created by deletion of the entire phosphopeptide from the phosphopeptide-bound complex. We consider this technique to be reasonable, as the crystal structures of the apo states from PDBID 2OGQ [13] and PDBID 3HIH [14] show no significant deviation from the holo states.

### Structure Preparation

The protein structures were initially prepared as follows. Atom coordinates for the protein, the phosphopeptide, and the water molecules were taken from the PDB. The hydrogen-atom positions for the protein and the water molecules were then built using the HBUILD facility of the CHARMM (version 34b1) program [27] with the CHARMM22 energy function [28]. Histidine residues were checked for protonation state manually. His382 was assigned as epsilon protonated and His 538 was assigned as positively charged for the phosphopeptide complex and as delta protonated for the peptide complex and the apo protein. All remaining histidines were assigned as delta protonated. The residues lysine, arginine, aspartate, glutamate, cysteine, and tyrosine were also analyzed to check their protonation state. There was no evidence of any unusual protonation states and thus all lysine and arginine residues were assigned as positively charged, all aspartate and glutamate residues were assigned as negatively charged, and all cysteine and tyrosine residues were assigned as neutral. The atomic charges of the standard residues were assigned from the CHARMM22 forcefield. The phosphate moiety from the phosphothreonine residue was assigned to be doubly deprotonated, as this is likely to be the dominant species at physiological pH of around 7.0, particularly when in close proximity to the positively charged binding site. The atomic charges of the dianionic phosphothreonine residues were assigned from the CHARMM27 forcefield, which is based on the charges of methylphosphate [29].

### Molecular Dynamics

**Figure 2:**
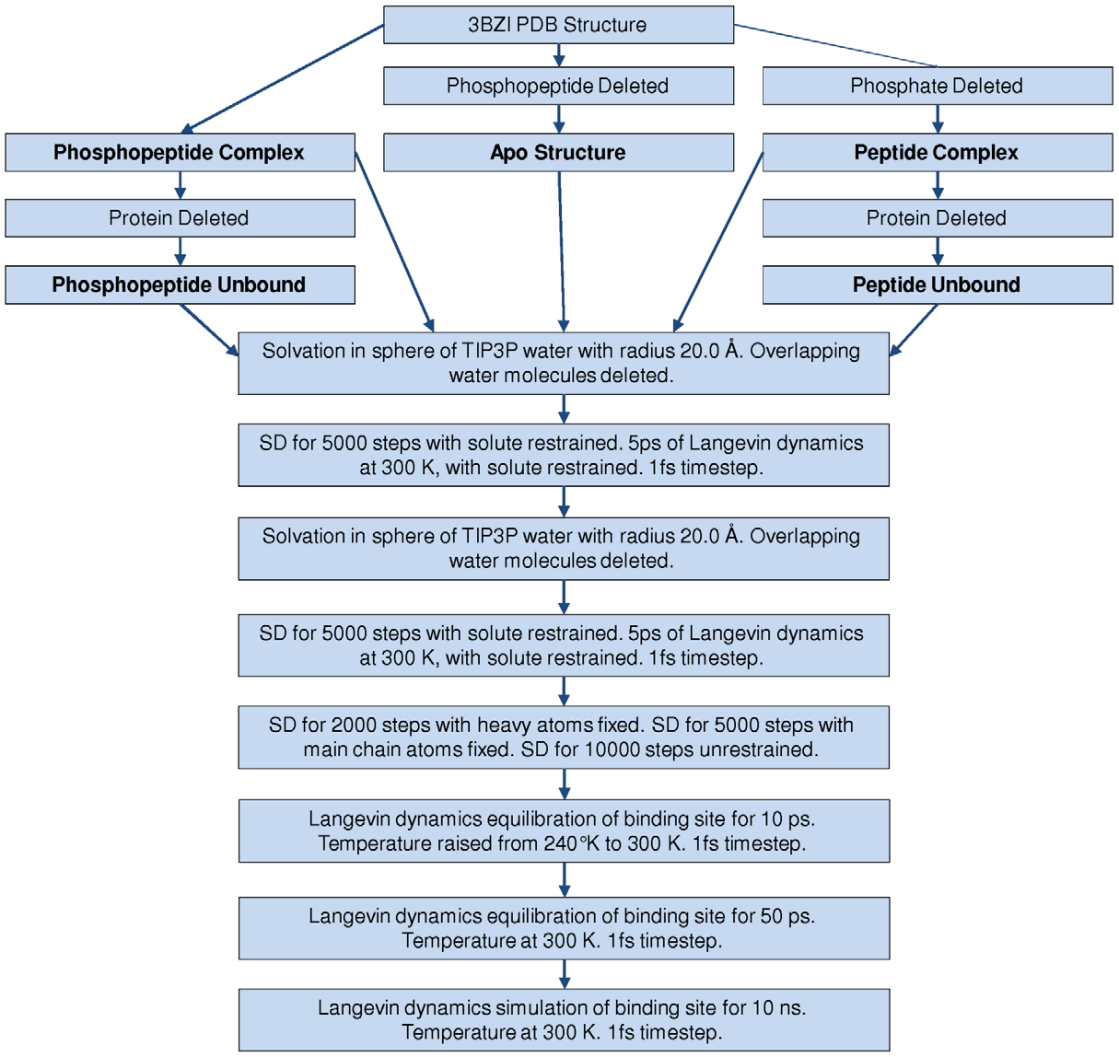
A schematic representation of the equilibration procedure. A schematic representation of the equilibration procedure used to prepare the systems for the MD simulations. The five systems that were simulated are highlighted in bold.

We performed MD simulations at 300 K on the apo state, the peptide complex and the phosphopeptide complex to investigate the dynamic nature of the interactions between the protein, the peptide or phosphopeptide and the water molecules. All three structures were prepared separately using the process schematically represented in Figure 2. In the first stage of preparation, the system was solvated with TIP3P water molecules [30] around the binding site. All the water molecules observed in the crystal structure were retained before solvating with the sphere of water molecules. Any water molecule overlapping with the protein, the crystal structure water molecules, the peptide or the phosphopeptide were removed. The solvent sphere of radius 20 Å was centred at the coordinates of the heavy atom centroid of the CDC25c phosphopeptide from 3BZI. The sphere completely enclosed the peptide and the binding site residues, extending at least 5.0 Å from the peptide or phosphopeptide. This assembly was partitioned into a 16 Å/20 Å reaction region/buffer region for stochastic boundary MD [27]. The solvent was then minimized by steepest descent (SD) for 5000 steps and then subjected to a 5 ps Langevin dynamics equilibration period at 300 K, during which the solute atom positions were fixed. The binding site was then repacked with TIP3P water molecules to fill any gaps. Any water molecule overlapping with the protein, the existing water molecules, the peptide or the phosphopeptide was removed. The solvent was again minimized by SD for 5000 steps and then subjected to another 5ps Langevin dynamics equilibration period at 300 K, during which the solute atom positions were fixed. The entire binding site was then subjected to minimisation by SD to allow the solute to adjust to the solvent for 2000 steps with heavy atoms fixed, for 5000 steps with main chain atoms fixed and then 10000 steps with no atom positions fixed. This was followed by a 10 ps Langevin dynamics equilibration period when the temperature was raised from 240 K to 300 K. Finally, the entire binding site was equilibrated at 300 K for 50 ps. We ensured that the system was brought to equilibrium before beginning the MD simulation by verifying that the system reached a point where the energy fluctuations were stable. Production simulations were then performed for 10.0 ns at 300 K.

During all CHARMM dynamics simulation, the positions of the main-chain heavy atoms were restrained using a 5.0 kcal/mol/Å^2^ harmonic force and the positions of the sidechain heavy atoms were restrained using a 1.0 kcal/mol/Å^2^ harmonic force. The MD simulations were performed using the CHARMM (version 34b1) program [27] with the CHARMM22 force field [28] and using the SHAKE [31] algorithm to constrain the bonds to hydrogen, allowing an MD time step of 1.0fs. The simulations were performed using a deformable boundary potential with a Langevin friction coefficient of 62.0 ps^−1^ applied to the water molecule oxygen atoms [32]. Electrostatic interactions were modelled with a uniform dielectric and a dielectric constant of 1.0 throughout the setup and production runs. This protocol has been used previously to analyse the dynamics of water molecules in the HSP90 system [33]. To explore the dynamics in the unbound state, simulations were performed on the peptide and phosphopeptide structures alone. In these cases the protein was deleted before preparing the system and the equilibration procedure in Figure 2 was employed.

### MM-PBSA Calculations

For the MM-PBSA calculations, we calculated the difference in free energy between the protein-ligand complex and the unbound protein plus the unbound ligand. MM-PBSA calculations were performed at intervals of 10 ps from each 10.0 ns run to yield 1000 snapshots. All of the water molecules were deleted and so not included explicitly in any of the MM-PBSA terms. For the electrostatics interactions a dielectric constant of 2.0 was employed, as this reflects the dielectric constant within the protein interior. The free energy change upon binding was calculated using the following equation:
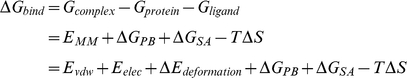
(1)E_MM_ is the molecular mechanics (MM) interaction energy between the receptor and the ligand, Δ*G_PB_* and Δ*G_SA_* are the electrostatic and non-polar contributions to desolvation upon ligand binding, respectively, and −TΔ*S* is the conformational entropy change, *E_vdw_* is the van der Waals interaction energy, *E_elec_* is the electrostatic interaction energy and Δ*E_deformation_* is the difference in internal energy between the bound state ligand and the unbound state ligand. This is termed the ligand deformation penalty. To calculate the ligand deformation penalty, we ran two separate MD simulations of the unbound ligands. MM-PBSA calculations were performed at intervals of 10 ps from each 2.0 ns run to yield 200 snapshots. The MM energies (Δ*E_MM_*) were calculated in CHARMM [27] for each snapshot. The deformation penalty of the ligand was considered by considering the MM energy of the peptide in both the bound state and unbound state simulations. This comprised electrostatic, van der Waals, and torsional contributions. We did not include the deformation penalty of the protein, as this involves taking the difference between the two large values of the protein internal energy. Even small errors in the individual energetic terms of these absolute internal energies can have a large impact on the relatively small energy differences and thus introduce large errors to the predicted binding energy [39]. The solvent accessible surface area (SASA) calculations were performed using CHARMM by calculating the change in surface area upon binding in Å^2^ multiplied by a constant value of 0.00542 kcal/mol plus the constant value of 0.92 kcal/mol.

To determine the key interactions between the phosphopeptide and the protein, we also calculated the per-residue contribution to the binding free energy for the phosphopeptide. Only sidechain atom contributions were included for each residue and the contributions from the mainchain atoms were calculated separately. The intramolecular interaction energies between each pair of residues was split in half and assigned evenly between the two residues. The Poisson-Boltzmann (PB) desolvation penalties were calculated with only the specific residue being considered. All other ligand atoms were deleted. As the desolvation is not pairwise additive, the sum of each desolvation piece differs to the total calculated with the complete ligand. The SASA terms were calculated for each residue without the SASA constant term. For all simulations, we also considered the standard error of the mean by dividing the simulation into 20 blocks of equal time and calculating the standard deviation of the individual components of the binding free energy.

### Poisson-Boltzmann Calculations

The PB calculations to determine Δ*G_PB_* were performed using a modified version of the DelPhi program [34]–[35] at a 129×129×129 grid resolution with focusing boundary conditions [36]. The molecular surface was used to represent the dielectric boundary, a dielectric constant of 2.0 was used for the molecular interior and a dielectric constant of 80.0 was used in the solvent region. An ionic strength of 0.145 M with a Stern layer of 2.0 Å was used for all PB calculations. For each snapshot, separate calculations were performed for the complex, the unbound protein and the unbound peptide. Protein atoms were assigned PARSE charges [37] before the DelPhi calculations. The Δ*G_PB_* for the Poisson-Boltzmann portion of the free energy changes were then calculated with the following equation:
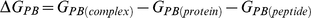
(2)G_pb(complex)_, G_pb(protein)_ and G_pb(peptide)_ are the PB solvation energies of the complex, the protein and the peptide respectively.

### Vibrational Entropy Changes

An estimate of the vibrational entropy change upon binding was performed, using normal mode analysis of the heavy atom fluctuations [38]. We only included the entropy change for the ligand, as simulation did not include the entire protein. Separate calculations were performed for the bound ligands and the unbound ligands and quasiharmonic analysis was used to estimate the vibrational entropies of the bound and unbound states. The VIBRAN module of the CHARMM program [27] was used to determine normal modes and normal-mode frequencies by diagonalisation of the force constant matrices. We used the entire 10.0 ns trajectories with a 20.0 fs timestep for the calculations. Water molecules were not included in this analysis. Translational and rotational motions were projected out from the dynamics trajectories by reorienting all the species using mass weighting. The frequencies of the vibrational modes for the heavy atoms were then computed at 298 K using a quasiharmonic approximation. The vibrational entropy of each system was then estimated from the vibrational frequencies [38].

### Static Binding Energy Calculations

Further binding energy calculations were performed on a static structure as a comparison. We began with the prepared phosphopeptide and peptide complexes. To adjust the complexes for use with CHARMM [27], the crystal structures were first subjected to a geometry optimization with the CHARMM22 energy function [28]. All receptor sidechains were harmonically restrained with a force constant of 1.0 kcal/mol/Å^2^ and all receptor backbone atoms were fixed. The minimization was performed for 1,000,000 steps using the adopted basis Newton-Raphson method. All water molecules were then deleted and the van der Waals, electrostatic and SASA terms were then calculated with CHARMM. To calculate the ligand deformation, the ligand structure was subjected to geometry optimization, separated from the receptor, for 1,000,000 steps. It was necessary to place harmonic restraints with a force constant of 1.0 kcal/mol/Å^2^ on all atoms to prevent the ligands from collapsing. The desolvation calculations were then performed using DelPhi [34]–[35] as described above. This can be considered a single-point MM-PBSA calculation.

### Energetics of Water Molecules at the Protein Surface

We performed additional analysis of the water molecules in the CHARMM MD simulations. Initially, we calculated the mean enthalpy of water molecules at specific sites at the binding interface. For each site, we analysed 1000 snapshots at 10.0 ps intervals across the 10.0 ns CHARMM simulation of the apo protein. For each snapshot, we considered every water molecule within 1.4 Å of each point and summed the MM energy. This allowed us to derive the mean value of the enthalpy for water molecules around each site. The relative energy with respect to bulk water was calculated by subtracting the mean enthalpy of a bulk water molecule. This was calculated using the MM energy from an MD simulation of a box of water molecules. We performed a 2.0ns simulation of a sphere of water molecules with 16Å/20Å reaction region/buffer region for stochastic boundary MD. To exclude water molecules at the surface of the sphere, only water molecules within 12.0 Å of the centre of the 20.0 Å sphere were included in the calculations. The mean value of the enthalpy of a bulk water molecule was calculated using 200 snapshots and found to be −18.5 kcal/mol. However, such an analysis only considers the enthalpic contribution to the free energy, ignoring the solvent entropy. We thus performed further calculations to explore the solvent enthalpy and entropy across the entire binding interface. There are a number of methods used to include the effects of solvent entropy on the free energy of water molecules. These include free-energy perturbation [39]–[40], thermodynamic integration [41] and inhomogeneous fluid solvation theory [42]. We chose to employ inhomogeneous fluid solvation theory, first described by Lazaridis [42] and implemented in Schrödinger's WaterMap software [43]–[45]. In this method, molecular dynamics simulations are analysed to cluster distinct hydration sites and assign an enthalpy and entropy to each one. The enthalpy is calculated as the average interaction energy over the simulation. The entropy is calculated by comparing the distributions of translations and orientations available to the water molecule in bulk water and at the surface.

We performed calculations on the apo state and the phosphopeptide complex. The WaterMap MD simulations were run with Desmond [46] using the OPLS_2005 force field [47]. We began with the prepared phosphopeptide complex and the apo protein. All water molecules from the crystal structure were deleted and TIP4P water molecules [48] were added with the System Builder module in Maestro. The solvated structure was then subjected to restrained minimisation using a force constant of 5.0 kcal/mol/Å^2^ on the solute heavy atoms. This was followed by a molecular dynamics simulation of 48.0 ps in which the temperature of the system was increased from 10 to 300 K. The harmonic restraints of 5.0 kcal/mol/Å^2^ on solute heavy atoms were retained. A preproduction simulation was then run at 300 K for 120.0 ps. Finally, the production simulations were run for 2.0 ns in the NPT ensemble at a temperature of 300 K and a pressure of 1 atm. The statistical analysis was performed using snapshots from the production simulation. Water molecules in the proximity of the binding site from 2000 equally spaced snapshots were clustered to form hydration sites. For each hydration site, the enthalpy was computed as the average non-bonded energy of each water molecule within the hydration site with the rest of the system. The excess entropy was computed by numerically integrating a local expansion of spatial and orientational correlation functions [44]. Only contributions from the first-order term of the expansion were included.

At this stage, we have not incorporated the effect of the solvent free energy on predictions of the total binding free energy of the phosphopeptide. A complete treatment of the energetics of the solvent would involve a consideration of all water molecules in the complex and the apo protein. It would also require a consideration of the unbound ligand. However, no prediction of the binding free energy is complete without such consideration. Protein surfaces can generate highly hydrophobic regions and this creates volumes of space where water molecules have unfavorable free energies. Filling these volumes with hydrophobic ligand atoms is a general mechanism to increase the ligand binding affinity. In some cases, there is a suggestion that water molecules will completely evacuate extremely hydrophobic cavities [43]. The creation of a small vacuum region leads to a large energetic penalty and filling such a cavity with a small molecule should greatly increase its affinity.

### Experimental Validation

To test the predictions made by the MM-PBSA calculations, the affinities of the CDC25c peptide and phosphopeptide were measured experimentally by fluorescence polarization (FP) [49] and by isothermal titration calorimetry (ITC). Human PLK1 amino acids 345 to 603 were amplified by PCR and cloned into the EcoRI and NotI sites of the bacterial expression vector pGEX6P-1, expressed in *E. Coli* and purified as previously described [12]. All peptides and phosphopeptides were synthesised using standard chemistry (Designer Bioscience Ltd., Cambridge, UK). The fluorescently labelled probe was the phosphopeptide sequence MAGPMQSpTPLNGAKK with N-terminal TAMRA. The peptide competitor was the sequence LLCSTPNGL and the phosphopeptide competitor was the sequence LLCSpTPNGL. FP measurements were carried out in a 384-well, low-volume, black, flat bottom polystyrene NBS microplate (Corning 3820) using a PHERAstar Plus plate reader (BMGLabtech). The final reaction volume of 45 µl contained 10nM labelled probe peptide, 35nM PBD and varying concentrations of competitor. FP values were obtained in millipolarisation units at an excitation wavelength of 540 nm and an emission wavelength of 590 nm, and were calculated in terms of percentage inhibitions. ITC measurements were performed using a VP-ITC microcalorimeter (MicroCal Inc.). The experiments consisted of injecting CDC25c peptide or CDC25c phosphopeptide at a concentration of 120 µM into a sample cell containing 12 µM of PBD in 50mM Hepes pH 7.4, 200mM NaCl, 1 mM EDTA, 1mM EGTA. Fifty injection of 4.5 µl of LLCSpTPNGL were performed with a spacing of 180 s using a reference power of 25mCal/s. Thirty injections of 8 µl of LLCSTPNGL were performed with a spacing of 240 s using reference power of 25mCal/s. All binding isotherms were analysed and graphed using Origin Software 7.5 (MicroCal Inc.).

## Results

Once the MD simulations were complete, they were subjected to a thorough analysis. The entire 10.0 ns of each trajectory was included in the analysis, as the systems had been pre-equilibrated. This was verified by two calculations. We calculated a time series of the root mean square fluctuation (RMSF) of the protein alpha carbon atoms. This value is stable over 10 ns for the apo protein, the peptide complex and the phosphopeptide complex (Figure S1). We also calculated a time series of the energetic components of the MM-PBSA binding free energy for the peptide complex and the phosphopeptide complex. The values are stable over 10 ns (Figures S2 and S3). For each trajectory, we considered the predicted free energy of binding for the peptide and the phosphopeptide, as well as the free energy of water molecules at the surface.

### MM-PBSA Calculations

The first calculation we performed was the prediction of the MM-PBSA binding free energy change for both the peptide and the phosphopeptide. The predicted binding free energy changes calculated by this method are presented in Table 1. The CDC25c phosphopeptide is predicted to bind with greater affinity than the corresponding peptide due to the more favourable binding enthalpy. As expected, the entropy changes of both ligands are unfavourable, due to the restriction on the ligand poses in the bound state. The calculations performed on the static structure are also presented in Table 1. Both the enthalpy and the change in enthalpy from the static calculations are similar to those predicted by the dynamic MM-PBSA calculations. This suggests that for this system, static calculations may be sufficient to predict relative binding enthalpies. However, such static calculations ignore the change in both the solute and the solvent entropy that we aimed to quantify with the MD simulations. To estimate the importance of each residue in the phosphopeptide, we analysed the per-residue contributions to the MM-PBSA binding enthalpy. The results are presented in Table 2. They highlight the importance of the phosphothreonine residue, which contributes over 30% of the binding enthalpy of the phosphopeptide. In fact, the phosphothreonine residue and the mainchain atoms together contribute over 75% of the binding enthalpy of the phosphopeptide. This begins to explains why the PBD does not discriminate strongly between different phosphopeptides, as in this case only a small contribution to the binding enthalpy is made by the non-phosphothreonine sidechains. The residue Leu1 makes a reasonable contribution to binding due to its strong van der Waals interactions with a hydrophobic surface. This is consistent with the experimental data from oriented peptide library screening, as leucine is one of the residues selected for at the −4 position [10], [14]. Residues Cys3 and Asn7 are predicted to make a small unfavourable contribution to binding. This is also consistent with the experimental data, as these residues are not selected at the −2 and +2 positions. However, residues Leu2 and Ser4 are predicted to make very little contribution to the binding enthalpy, but are selected at positions −3 and −1 respectively [9]–[10]. The importance of these residues is revealed by considering the interactions of the solvent in the later sections.

**Table 1:** Predicted binding free energy change for the CDC25c phosphopeptide and peptide from dynamic and static structure calculations.

	CDC25c Phosphopeptide (MMPBSA)	CDC25c Peptide (MMPBSA)	CDC25c Phosphopeptide (Static)	CDC25c Peptide (Static)
VDW (kcal/mol)	−41.3±1.2	−36.0±0.8	−56.0	−53.0
Electrostatic Interaction (kcal/mol)	*−187.5±3.4*	*−25.6±1.2*	−241.0	−72.6
Desolvation Penalty (kcal/mol)	*208.3±2.6*	*50.3±1.7*	252.4	87.7
SASA(kcal/mol)	*−2.8±0.0*	*−2.7±0.0*	−5.6	−5.1
Ligand Deformation (kcal/mol)	−9.3	−7.9	5.9	10.1
**Δ** ***H*** ** (kcal/mol)**	**−32.7**	**−21.9**	**−44.2**	**−32.9**
**ΔΔ** ***H*** ** (kcal/mol)**		**10.8**		**11.3**
Unbound Peptide TS (kcal/mol)	71.8±1.1	62.5±1.0	NA	NA
Bound Peptide TS (kcal/mol)	58.1±0.7	53.3±1.1	NA	NA
**−TΔ** ***S*** ** Peptide (kcal/mol)**	**13.7**	**9.2**	**NA**	**NA**
**−ΔTΔ** ***S*** ** Peptide (kcal/mol)**		**−4.5**		**NA**
**Δ** ***G*** ** (kcal/mol)**	**−19.0**	**−12.7**	**NA**	**NA**
**ΔΔ** ***G*** ** (kcal/mol)**		**6.2**		**NA**

The predicted binding free energy change for the CDC25c phosphopeptide and the CDC25c peptide from the MM-PBSA and static structure calculations. The separate contributions to the binding free energy change are also reported. The ligand deformation is the difference between the internal energy of the ligand in the bound and the unbound states. The entropic contribution to the binding free energy from the peptide is the difference between the entropy in the bound and unbound states calculated by normal mode analysis. The standard errors of the mean are estimated from twenty equally sized blocks.

**Table 2:** Per-residue contributions to the predicted MM-PBSA binding free energy change for the CDC25c phosphopeptide (kcal/mol).

Residue	VDW Interaction	Electrostatic Interaction	Ligand Deformation	Desolvation	SASA	Total	Percent Total
*Mainchain*	−20.5±0.4	−22.3±1.0	−2.3	37.1±1.1	−1.9±0.0	−9.7	**42.5**
*Leu 1 (−4)*	−4.3±0.4	0.1±0.0	−1.0	1.5±0.1	−0.2±0.0	−4.0	**17.3**
*Leu 2 (−3)*	−2.8±0.3	0.2±0.0	−0.8	3.1±0.3	−0.2±0.0	−0.5	**2.3**
*Cys 3 (−2)*	−0.5±0.5	0.1±0.1	−0.9	2.7±0.1	−0.2±0.0	1.2	**−5.1**
*Ser 4 (−1)*	−4.2±0.4	0±0.4	−2.5	6.5±0.3	−0.1±0.0	−0.3	**1.5**
*pThr 5 (0)*	−2.5±1.0	−162.0±3.7	−0.7	157.8±2.6	−0.4±0.0	−7.8	**34.1**
*Pro 6 (+1)*	−2.0±0.1	3.1±0.1	−1.8	−1.3±0.1	0.0±0.0	−1.9	**8.4**
*Asn 7 (+2)*	−4.5±0.2	−6.7±0.2	0.6	11.0±0.2	−0.1±0.0	0.2	**−1.0**
*Totals*	−41.3±1.2	−187.5±3.4	−9.3	218.3±2.6	−3.1±0.0	−22.9	**100.0**

The predicted MM-PBSA binding free energy change for the CDC25c phosphopeptide, split into per-residue contributions, and the percentage of each contribution to the total binding free energy. The standard errors of the mean are estimated from twenty equally sized blocks.

### Experimental Validation

**Figure 3:**
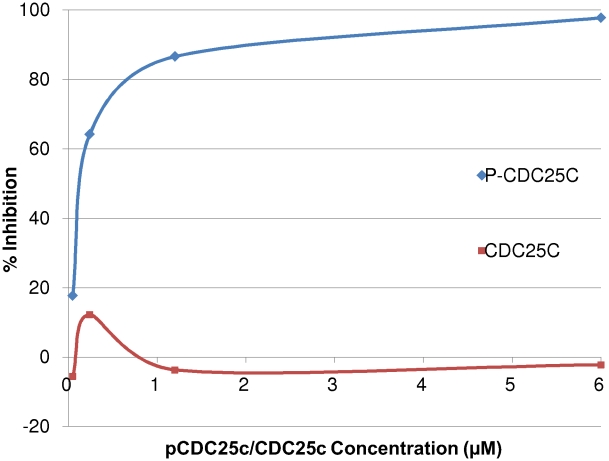
The results of CDC25c peptide and CDC25c phosphopeptide binding to the PBD by FP. The effect of unlabelled CDC25c peptide and CDC25c phosphopeptide on the binding of the PBD to the TAMRA labelled consensus phosphopeptide sequence in the FP Assay. The phosphopeptide or peptide concentrations are reported on the x axis and the percentage inhibitions are reported on the y axis. The inhibition curves are coloured blue for the phosphopeptide and red for the peptide.

**Figure 4:**
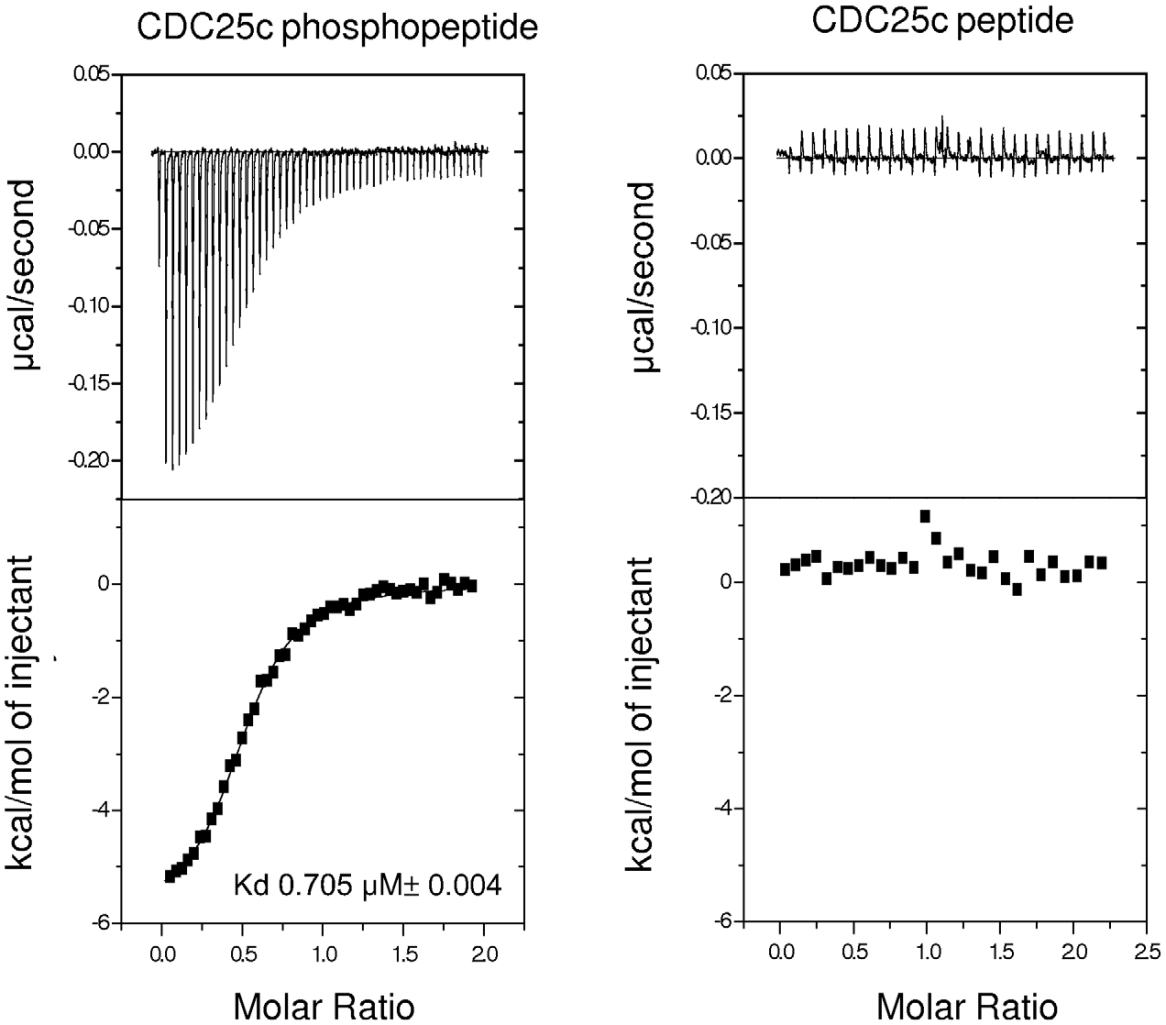
The results of CDC25c peptide and CDC25c phosphopeptide binding to the PBD by ITC. ITC data for the binding of CDC25c peptide and CDC25c phosphopeptide to the PBD of PLK1. The ITC traces and the determined thermodynamic parameters of binding are shown. The CDC25c peptide displays no detectable binding. The binding of the CDC25c phosphopeptide can be fit to a Kd of 0.705 µM. Errors in thermodynamic parameters are derived from the fitting error after repeating the experiment at least three times.

The MM-PBSA calculations predict that the CDC25c phosphopeptide will bind to the PBD with higher affinity than the CDC25c peptide. We tested this prediction experimentally using an FP assay and by ITC. We measured the effect of both the peptide and the phosphopeptide on binding of a fluorescently tagged phosphopeptide. The FP results can be seen in Figure 3 and show that, as predicted, the CDC25c phosphopeptide has a significantly higher affinity than the CDC25c peptide, which showed no measurable binding. We also measured the binding of the CDC25c peptide and the CDC25c phosphopeptide by ITC. The ITC data can be seen in Figure 4 and confirms that the CDC25c peptide shows no detectable binding and the CDC25c phosphopeptide binds with a measured affinity of 0.705 µM. These results are consistent with the biological function of PLK1 and with prior experimental work on other phosphopeptides, but have not previously been explained quantitatively.

### Interfacial Water Molecules

**Figure 5:**
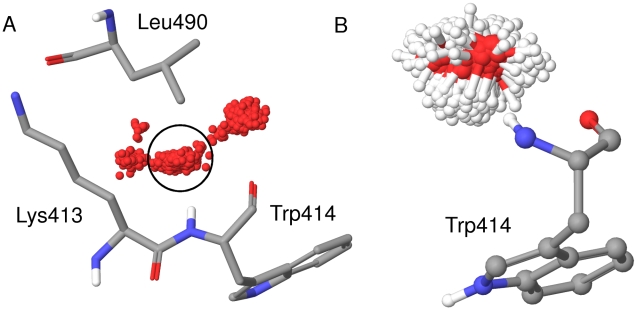
Snapshots of water molecules during the 10.0ns simulation. Translational and orientational ordering of water molecules at specific sites during the 10.0ns simulation. (a) 1000 snapshots of the oxygen atom for one water molecule, illustrating the presence of distinct hydration sights. PBD residues Lys 413, Trp414 and Leu 490 are displayed as atom coloured sticks and labelled. (b) Snapshots of any water molecule coordinating to the backbone amide of Trp414 during the simulation, illustrating the restricted rotational freedom of water molecules at this position. PBD residue Trp414 is displayed as atom coloured balls and sticks and labeled.

In order to understand the dynamic nature of the system, we looked at the trajectory of each individual water molecule in each simulation and calculated the RMSF of the oxygen atom from its mean position. The water molecules in the sphere of water molecules are highly mobile, with a mean RMSF of 11.5 Å. No water molecule has an RMSF below 2.0 Å. However, in all three protein simulations there are a large number of water molecules near the protein surface with an RMSF below 0.5 Å. It is clear that even in the apo state, water molecules are fixed to some degree at the surface. It is possible that a larger sphere of water molecules or a longer timescale is needed to accurately model the mobility of these surface water molecules. However, the conclusion is supported by analysis of the water molecules in the apo crystal structures, which have B factors similar to the protein residues. A closer examination of individual water molecules across the simulation reveals the presence of distinct hydration sites at the surface, formed by hydrogen bonding interactions with the protein. Figure 5a shows four such hydration sites, occupied by one water molecule during the course of the 10.0ns simulation of the apo protein. Water molecules at the circled site form a hydrogen bond to the backbone amide of residue Trp414. Water molecules in this hydration site are expelled upon ligand binding and replaced by the serine residue at the −2 position of the consensus sequence. We estimated the enthalpy of water molecules at this site with respect to bulk water for each of the 1000 snapshots of the apo protein. We placed the hydration site at a position 3.0 Å along the amide nitrogen to amide hydrogen bond vector. For each snapshot, we considered every water molecule within 1.4 Å of the site, representing the idealized radius of a water molecule. There is a water molecule within this sphere in 99% of snapshots, whilst the average enthalpy of water molecules within the sites with respect to bulk water is +1.3±1.5 kcal/mol. Expulsion of water molecules from this site should thus provide an enthalpic bonus upon ligand binding. However, water molecules at the circled hydration site show marked translational and orientational ordering, as shown in Figures 5b. This corresponds to an unfavourable entropy with respect to bulk water and should lead to an entropic bonus to ligand binding upon expulsion of a water from this hydration site. We thus employed inhomogeneous fluid solvation theory, implemented in Schrodinger's WaterMap, to explore the enthalpy and entropy of water molecules at this surface.

### Free Energy Calculations

**Figure 6:**
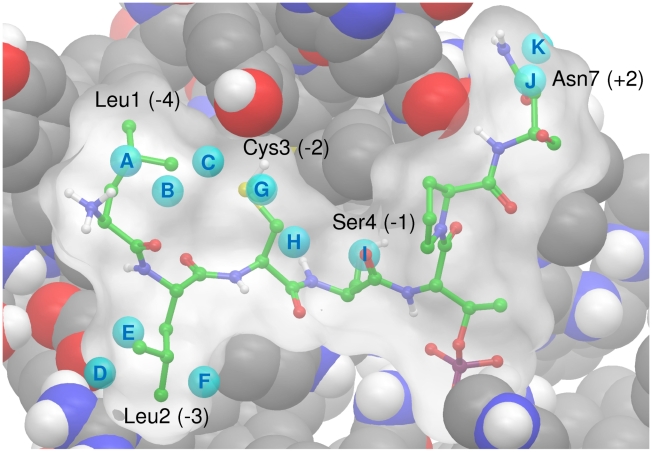
An overlay of the CDC25c phosphopeptide on a subset of the hydration sites from a simulation of the apo state. A subset of the hydration sites identified by WaterMap from the apo state simulation that overlap with the phosphopeptide. Hydration sites are displayed as cyan balls and labeled with letters A–K. The overlayed phosphopeptide is displayed as atom colored balls and sticks with a molecular surface in white. Residues Leu1, Leu2, Cys3, Ser4 and Asn7 are labeled. PBD binding site residues are displayed in atom coloured CPK.

Analysis of the WaterMap results highlights a number of important points. Eleven hydration sites from the apo protein that overlap with the phosphopeptide sidechains are shown in Figure 6. Details of the enthalpy and entropy of the hydration sites are given in Table 3. It is clear that phosphopeptide binding expels a number of unsatisfied water molecules from hydration sites on the surface of the PBD, including the site I, which was identified by the CHARMM simulation. Water molecules in these hydration sites do not make their full complement of four hydrogen bonds, the majority have an unfavourable enthalpy with respect to bulk water and all eleven have an unfavourable entropy. The WaterMap calculations suggest that a water molecule at hydration site I is particularly unfavourable with respect to bulk water and displacement by phosphopeptide binding is predicted to yield 3.56 kcal/mol in binding free energy. This explains the importance of serine at the −1 position in this sequence. Large residues are unable to fit in this small hydrophobic cavity and the smaller residues glycine or alanine would not displace a water molecule from this site. In fact, they would likely further destabilize it due to desolvation. In fact, expulsion of surface water molecules is predicted to yield over 3.0 kcal/mol for each of the residues Leu1, Leu2, Cys3, Ser4 and Asn7 as shown in Table 3. The total contribution to the binding free energy of expelling all eleven water molecules is predicted to be 20.26 kcal/mol. 13.85 kcal/mol of this is derived from a favorable change in the solvent entropy. This is of great significance, as the binding free energy from the MM-PBSA calculation is only −19.0 kcal/mol. It is also significant, as it explains the requirement for hydrophobic residues at the −4, −3 and +2 positions. Any large residue at one of these positions can extend across the protein surface and displace ordered water molecules, yielding an entropic bonus to the binding free energy. The consensus phosphopeptide for the PLK1 PBD thus contains a number of non-specific hydrophobic residues. It is interesting to note that the N-terminal proline residues of the optimal synthetic phosphopeptide PMQSpTPL [10] and the high-affinity short phosphopeptide PLHSpT [14] both overlap with hydration sites D, E and F as well as further unfavourable hydration sites (data not shown).

**Table 3:** Energetic analysis of hydration sites where a water molecule is displaced by CDC25c phosphopeptide binding.

Residue	Site	Occupancy	Mean HBs	Δ*H*	−TΔ*S*	Δ*G*	ResidueΔ*H*	Residue −TΔ*S*	ResidueΔ*G*
*Leu 1*	A	0.30	2.60	1.57	0.85	2.42			
*Leu 1*	B	0.30	3.18	−0.05	0.85	0.80			
*Leu 1*	C	0.28	3.02	1.22	0.73	1.95	2.74	2.43	5.17
*Leu 2*	D	0.29	3.60	0.31	0.81	1.12			
*Leu 2*	E	0.73	2.76	−2.50	2.46	−0.04			
*Leu 2*	F	0.36	3.06	1.50	1.03	2.53	−0.69	4.30	3.61
*Cys 3*	G	0.33	3.06	2.01	0.85	2.86			
*Cys 3*	H	0.58	2.64	−0.58	1.73	1.15	1.43	2.58	4.01
*Ser 4*	I	0.84	2.20	0.80	2.76	3.56	0.80	2.76	3.56
*Asn 7*	J	0.32	2.99	1.22	0.86	2.08			
*Asn 7*	K	0.32	3.04	0.91	0.92	1.83	2.13	1.78	3.91
***Total***							**6.41**	**13.85**	**20.26**

Details of eleven hydration sites from the apo protein surface that overlap with sidechains of the bound phosphopeptide. The occupancy, mean number of hydrogen bonds (Mean HBs), change in enthalpy, entropy and free energy are relative to bulk water. The sites are labelled A–K and are displayed in Figure 6. The enthalpy, entropy and free energy are weighted by the occupancy of the site.

**Figure 7:**
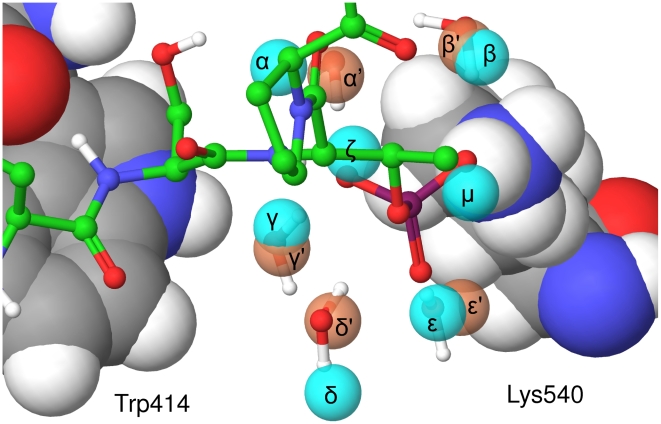
Hydration sites surrounding the phosphate group in the apo state and the phosphopeptide complex. Hydration sites identified by WaterMap that surround the phosphate group. The hydration sites from the apo state simulation are displayed as cyan balls and labeled α-μ. The hydration sites from the complex simulation are displayed as pink balls and labeled α′–ε′. The phosphopeptide and five water molecule from the crystal structure are displayed as atom colored balls and sticks. PBD binding site residues Trp414 and Lys540 are displayed in atom coloured CPK and labelled.

The second role of water molecules at this interface is more subtle. In the complex, the presence of the charged phosphate group greatly affects water molecules in the seven hydration sites shown in Figure 7. Water molecules ζ and η are expelled upon ligand binding and this is predicted to have a net unfavourable contribution to binding, as detailed in Table 4. However, water molecules α, β, γ, δ, and ε are all present in the apo and the bound states. These five water molecules are also observed in the crystal structure of 3BZI with low B factors. The positions from the crystal structure of the complex are also shown in Figure 7 and are very close to the predicted hydration site, which are coloured red. The five water molecules are pre-ordered in the apo state, with an unfavourable entropy with respect to bulk water, but are pre-ordered in the correct geometry to coordinate the phosphate. Thus, whilst phosphopeptide binding further orders these water molecules, it also provides them with a huge enthalpic bonus, as shown in Table 4. In fact, stabilizing these five water molecules is predicted to yield −17.86 kcal/mol. Thus, stabilization provides a greater free energy bonus than displacement, which would only yield −11.71 kcal/mol. This effect of stabilizing ordered water molecules has not previously been quantified and these results suggest that this can represent a significant component of binding free energies.

**Table 4:** Energetic analysis of hydration sites where a water molecule is stabilized by CDC25c phosphopeptide binding.

Site	Occupancy Apo/Bound	Δ*H* Apo	Δ*H* Bound	−TΔ*S* Apo	−TΔ*S* Bound	Δ*G* Apo	Δ*G* Bound	ΔΔ*H* Bind	ΔTΔ*S* Bind	ΔΔ*G* Bind
*α*	0.86/1.00	−1.13	−7.80	2.94	5.11	1.81	−2.69	−6.67	2.17	−4.50
*β*	0.45/1.00	1.14	−5.10	1.33	4.41	2.47	−0.69	−6.24	3.08	−3.16
*γ*	0.96/1.00	−1.52	−9.89	3.39	5.66	1.87	−4.23	−8.37	2.27	−6.10
*δ*	0.92/1.00	0.65	−3.07	3.63	5.58	4.28	2.51	−3.72	1.95	−1.77
*ε*	0.42/0.99	0.11	−4.91	1.17	3.30	1.28	−0.51	−5.02	2.13	−1.79
*ζ*	0.42/0.00	−0.95	NA	1.29	NA	0.34	NA	0.95	−1.29	−0.34
*μ*	0.60/0.00	−2.62	NA	1.82	NA	−0.80	NA	2.62	−1.82	0.80
***Sum***						**11.25**	**−5.61**	**−26.45**	**8.49**	**−16.86**

Details of seven hydration sites from the apo protein surface in the proximity of the phosphate group. The occupancy, change in enthalpy, entropy and free energy are relative to bulk water. The changes in the energy changes upon binding are the difference between the apo and the bound states. The sites are labelled α-μ and are displayed in Figure 7. The enthalpy, entropy and free energy are weighted by the occupancy of the site.

## Discussion

Our simulations make a number of predictions. The first is that the CDC25c phosphopeptide will bind to the PBD with higher affinity than the unmodified CDC25c peptide. This prediction was confirmed by the results of the FP and ITC experiments, in which the phosphopeptide was found to bind strongly with a Kd of 0.705 µM but the peptide showed no detectable binding. It is also consistent with the biological understanding of PLK1, which binds phosphopeptides in vivo, and agrees with recent work showing that the phosphopeptide LHSpTAI binds to the PBD of PLK1 with a Kd of 0.247 µM but the peptide LHSTAI shows no detectable binding [14]. The per-residue contributions to the enthalpy predict that the main determinants of binding are the mainchain hydrogen bonding interactions and the negatively charged phosphate group interacting with the electropositive bath of water molecules around His538 and Lys540. This is illustrated by the contribution of over 75% to the binding enthalpy made by the mainchain atoms and the phosphothreonine residue alone. The sidechains of residues at the −3, −2, −1, +1 and +2 positions are predicted to make only a small contribution to the binding enthalpy. This explains why peptides do not bind and also explains the lack of selectivity, with many diverse phosphopeptide sequences binding. It is important to note that the per residue predictions will only correspond to the biological importance of these residues if the phosphopeptide adopts the same orientation as the phosphoprotein it represents. Specifically, residues at the −4 and −3 positions appear able to adopt very different orientations in different phosphopeptides whereas this may not be true for the corresponding phosphoproteins due to secondary structure constraints.

However, the simulations also highlight the importance of water molecules at this interface, which MM-PBSA calculations do not explicitly consider. As noted in the introduction, bridging water molecules appear to be very important in mediating interactions between the PBD and its target phosphopeptides. Initial analysis with CHARMM identifies distinct hydration sites on the protein surface and predicts water molecules are enthalpically unfavourable with respect to bulk water. Visual inspection suggests that there is significant ordering of these water molecules and thus that there will be a change in solvent entropy upon ligand binding. We thus employed inhomogeneous fluid solvation theory to consider solvent enthalpy and entropy. It is important to note that small inaccuracies in calculations of free energies for individual water molecules will cause major inaccuracies in overall predictions, as there may be a large number of interfacial water molecules and any errors are multiplied. However, in cases where there are a large number of interfacial water molecules, such calculations are very important and may dominate the free energies calculated by MM-PBSA or MM-GBSA methods. The overall contribution of the solvent free energy change should be termed the hydric effect and not the hydrophobic effect, because displacement of some water molecules contributes positively to binding and displacement of others contributes negatively to binding.

The WaterMap analysis of hydration sites on the surface of the PBD predicts that water molecules have two distinct effects on the binding in this system. Firstly, ligand binding expels water molecules from hydration sites that are both enthalpically and entropically unfavourable with respect to bulk water. In particular, the key Ser4 residue from the phosphopeptide displaces a highly unfavourable water molecule in a hydrophobic cavity and this explains its importance. At other positions in the sequence, expulsion of water molecules from hydration sites on the hydrophobic surface provides a bonus to the binding free energy that is non-specific, as any large residue would have a similar effect. The promiscuous binding of the PBD due to elimination of ordered water molecules from the surface is consistent with suggestions that the hydrophobic effect is a general mechanism to provide affinity, but specificity must be achieved by electrostatic complementarity. Such broad specificity for substrates is also observed for the oligopeptide binding protein OppA and this behaviour has been attributed to the presence of bridging water molecules [50]. It would be interesting to study this system to analyze the importance of water elimination and water stabilization in providing a general mechanism for broad specificity. However, water molecules are also found at sites with a much narrower specificity such as the SRC SH2 domain [51] and the L-arabinose-binding protein [52]. Interfacial water molecules are thus able to elicit opposite effects of both broad and narrow specificity [53]–[55]. This highlights the importance of explicitly considering water molecules within each particular system. The second predicted effect on the binding free energy is that the phosphate group of the ligand strongly stabilizes a number of water molecules that are highly ordered at the apo protein surface but are enthalpically unfavorable. This stabilization is an effect that has not previously been described and quantified and it will be interesting to examine other systems to determine whether this is a common phenomenon.

A WaterMap analysis performed on the PBD of PLK1 highlights hydration sites containing ordered water molecules where ligand binding should lead to a favorable free energy change. The binding conformations of a number of phosphopeptides determined from the crystal structures overlay a number of such hydrophobic sites and the hydrophobic effect is likely to be one of the major determinants of binding in these systems. In particular, the backbone amide and carbonyl groups of Trp414 create two highly unfavourable hydration sites H and I in Figure 6 that are trapped between the hydrophobic sidechains of Trp414 and Leu490. Hydrophobically enclosed correlated hydrogen bonds such as these have been identified previously as a key determinant of binding [43]. Developing small molecule inhibitors that specifically target these hydration sites is likely to yield high-affinity binding for small molecule inhibitors of the interaction between the phosphopeptide and the PBD. The observation that PLK1 is over-expressed in human cancer has led to the development of several PLK1 inhibitors targeting the ATPase activity for cancer therapy [56]–[61]. However, the genetic inactivation of PLK1 can trigger mitotic arrest and apoptosis in a range of cells and tissues [62]–[63], raising the possibility that more subtle means to interrupt abnormal PLK1 function may offer an improved therapeutic window. A number of small molecule inhibitors of the PBD of PLK1 have been reported to date [64]–[65]. Interestingly, molecular docking results on the molecule purpurogallin [66] suggest that it binds in the phosphopeptide binding groove and visual inspection suggests that binding would displace the three highly unfavourable water molecules I, α and γ. In this light, our observations concerning the recognition of phosphopeptide substrates by the PLK1 PBD may open new perspectives for the design of inhibitors that inhibit the activity of PLK1 towards specific substrates. Thus, the analysis performed in this study is likely to be useful to drug development efforts. It is important to note that affinity gain due to displacement of unsatisfied water molecules would not be modelled explicitly by traditional scoring functions because they do not consider the apo state. The hydrophobic effect is known to be a key determinant of binding and is one of the major driving forces of binding. This is one of the major deficiencies in current scoring functions, particularly when applied to protein-protein interactions. An accurate treatment of the free energy of interfacial water molecules would be a significant improvement to current approaches and may prove crucial for accurately predicting binding free energies at protein-protein interfaces. A complete treatment will require an analysis of the unbound states of the ligand and the protein as well as the bound complex.

In conclusion, our results highlight the importance of water molecules in the binding between CDC25c and the PLK1-PBD and suggest that an explicit consideration of solvent is vital when studying protein-protein interactions. The work presented here adds to the growing literature showing that the free energy of interfacial water molecules is different from the free energy of bulk water molecules and that this can make a major contribution to binding. Finally, our analysis identifies a number of sites where small molecule inhibitors should gain affinity by displacing or stabilizing highly ordered water molecules.

## Supporting Information

Figure S1 Time series of the RMSF of the backbone alpha carbons.
(0.19 MB TIF)

Figure S2 Time series of the MM-PBSA energy components for the phosphopeptide.
(0.14 MB TIF)

Figure S3 Time series of the MM-PBSA energy components for the peptide.
(0.72 MB TIF)
