# Supplementary figures and images for: Computational Analysis of Phosphopeptide Binding to the Polo-Box Domain of the Mitotic Kinase PLK1 Using Molecular Dynamics Simulation
Source: PLoS Comput Biol. 2010 Aug 12;6(8):e1000880. doi: 10.1371/journal.pcbi.1000880 (PMC2920843; doi:10.1371/journal.pcbi.1000880)

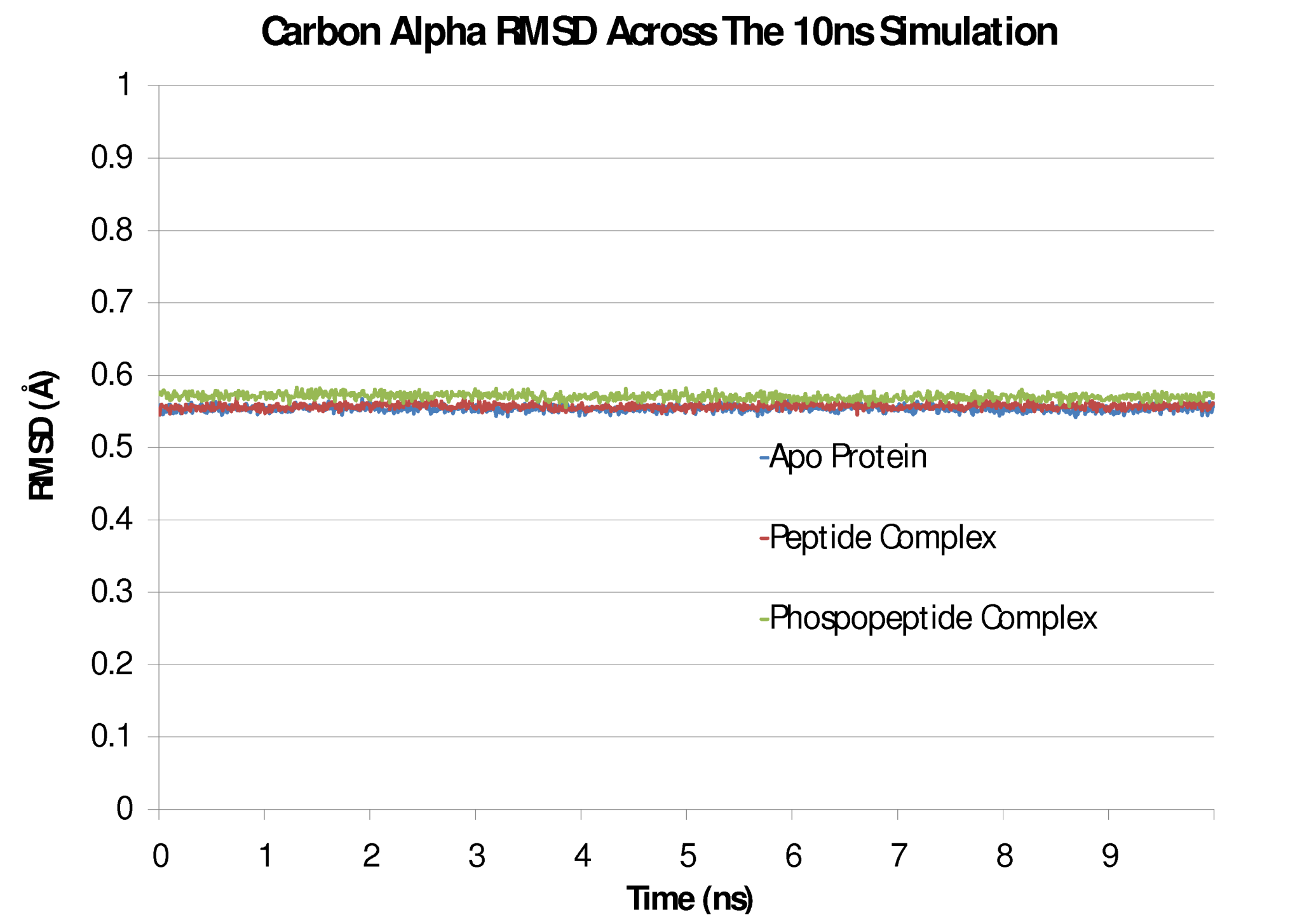

Supplement: Figure S1 — Time series of the RMSF of the backbone alpha carbons. (0.19 MB TIF) [file pcbi.1000880.s001.tif]

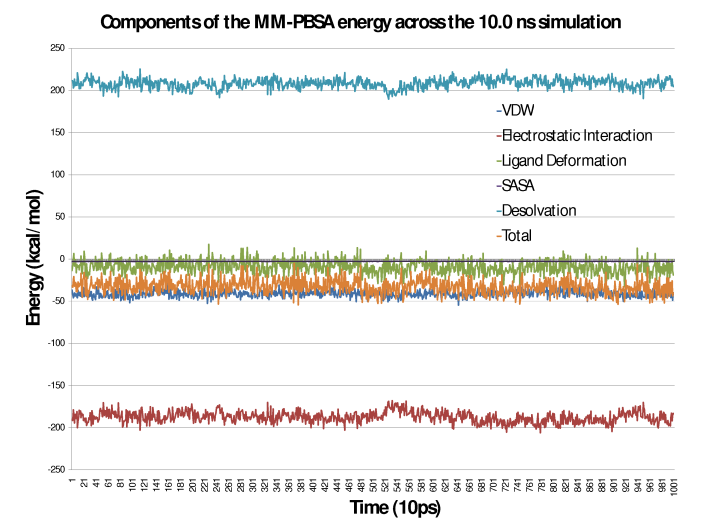

Supplement: Figure S2 — Time series of the MM-PBSA energy components for the phosphopeptide. (0.14 MB TIF) [file pcbi.1000880.s002.tif]

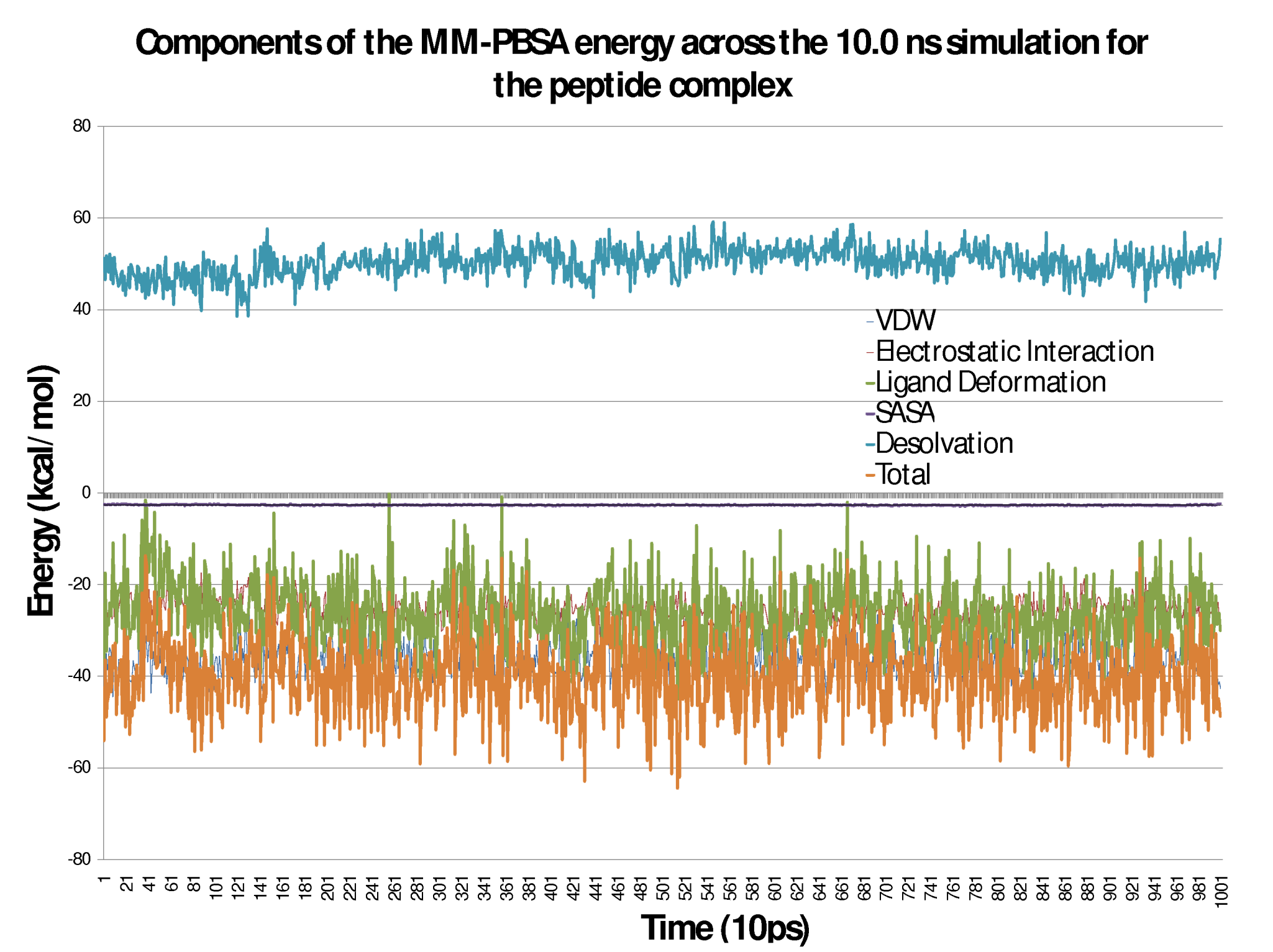

Supplement: Figure S3 — Time series of the MM-PBSA energy components for the peptide. (0.72 MB TIF) [file pcbi.1000880.s003.tif]
